# The prevalence of human papillomavirus and bacterial vaginosis among young women in China: a cross-sectional study

**DOI:** 10.1186/s12905-021-01504-0

**Published:** 2021-12-09

**Authors:** Wenyu Lin, Qiaoyu Zhang, Yaojia Chen, Lihua Chen, Binhua Dong, Pengming Sun

**Affiliations:** 1 Department of Gynecology, Fujian Maternity and Child Health Hospital, Affiliated Hospital of Fujian Medical University, Fuzhou, China; 2 Laboratory of Gynecologic Oncology, Department of Gynecology, Fujian Maternity and Child Health Hospital, Affiliated Hospital of Fujian Medical University, No, 18 Rd. Daoshan, Fuzhou, 350001 Fujian People’s Republic of China; 3 Fujian Key Laboratory of Women and Children’s Critical Diseases Research, Fujian Maternity and Child Health Hospital, Affiliated Hospital of Fujian Medical University, Fuzhou, 350001 China

**Keywords:** HPV infection, Bacterial vaginosis, Young women, Fujian population

## Abstract

**Background:**

The natural history of human papillomavirus (HPV) is influenced by vaginal microenvironment disorders, such as bacterial vaginosis (BV). The objective of this study was to assess the epidemiology of HPV combined with BV prevalence among Chinese women aged 20–35 years.

**Methods:**

A total of 2000 sexually active women aged 20–35 years voluntarily enrolled in this study and underwent a ThinPrep cytologic test and PCR-reverse dot blot human papillomavirus genotyping (PCR-RDB HPV test). BV was diagnosed if clue cells were observed (20% more than epithelial cells).

**Results:**

The overall HPV infection rate in this population was 16.2% (324/2000). Compared with HPV-negative individuals, BV prevalence was higher in the High-risk human papillomavirus (HR-HPV) (5.9% vs. 3.1%, *P* < 0.001). BV and HPV-51, -52 infection were more commonly associated with each other. In patients with cervical lesions (≥ CIN 1), the BV prevalence rate was higher than in patients with negative for intraepithelial lesion or malignancy (NILM) (11.9% vs. 3.8%, *P* = 0.002).

**Conclusion:**

BV was found to be related to HPV-51, -52 infections and cervical lesions. To better manage HPV infected population, more attention should be paid to the prevention and proper treatment of BV.

## Introduction

Cervical cancer is the fourth most frequently occurring cancer among women worldwide and is particularly common in resource-limited countries. Approximately, 311,365 cervical cancer-related deaths occurred in 2018 [1]. Persistent HPV infection is the main risk factor for the development of high-grade cervical lesions and even cervical cancer. The peak of HPV infection in girls is approximately 20 years of age. The natural history of HPV is influenced by several factors, such as bacterial vaginosis (BV). Previously, Menon et al. [2] suggested an association between BV and HR-HPV and the need for sexually transmitted disease management within a cervical cancer prevention program, though a relationship between cervical lesions and BV was not demonstrated. A study in Beijing suggested that HPV with BV infection possibly increasing the incidence of cervical intraepithelial neoplasia (CIN) and cervical cancer [3]. The association between BV and cervical lesions varies according to the characteristics of the study population, such as geography, race, different detection methods and other factors. BV prevalence rates differ among cities and even within a similar study population, ranging from 8 to 75%. Although many studies have been performed, data related to BV, HPV and cervical lesions for China are limited, and the relationship between BV and cervical lesions remains controversial.

There is a high prevalence rate for both BV and HPV among sexually active women, with a low cervical lesion rate [4]. Although cervical cancer mostly occurs in women aged 30–39 and 60–69 years, one study suggested that cervical cancer in the young tends to be more aggressive [5]. Cervical cancer screening is an effective measure to decrease the burden of cervical cancer. However, many factors hinder the success of cervical cancer screening programs, such as limited knowledge of HPV and the economic burden caused by screening, particularly in resource-limited countries. China is the largest resource-limited country, and compared with regular cervical cancer screening, vaccination against HPV may be particularly useful and effective. In mainland China, Cervarix (GlaxoSmithKline, Wales) was approved in 2016 for females aged 9–45 years, in 2017, Gardasil (Merck Inc, Whitehouse Station) was launched for women aged 20–45 years, and Gardasil-9 was rolled out in 2018 in several regions of China (partly in Fujian) for women aged 16–26 years. Despite the numerous published studies focusing on HPV in recent years, only a few studies are focusing on HPV among women aged 20–35 years, considering that HPV prevalence is relatively higher in Mainland China [6]. Nonetheless, the vaccination rate remains low and all available vaccines are currently developed based on epidemiological data from Western countries. Therefore, these vaccines may not archive the desired efficiency and it is essential to develop policies on how to apply HPV vaccination to cervical cancer control programs in targeting Chinese women. The vaccine had not been introduced in China before this study. Data on the prevalence and distribution of HPV genotypes in young populations are important both for vaccination campaigns and for monitoring the impact of vaccination on the prevalence of HPV types.

Our purpose was to assess the epidemiology of HPV and BV prevalence, analyze cytological and histological statuses and evaluate their association, thus providing a suitable protection program reference for the young Chinese population.

## Materials and methods

### Study design and participants

**Fig. 1:**
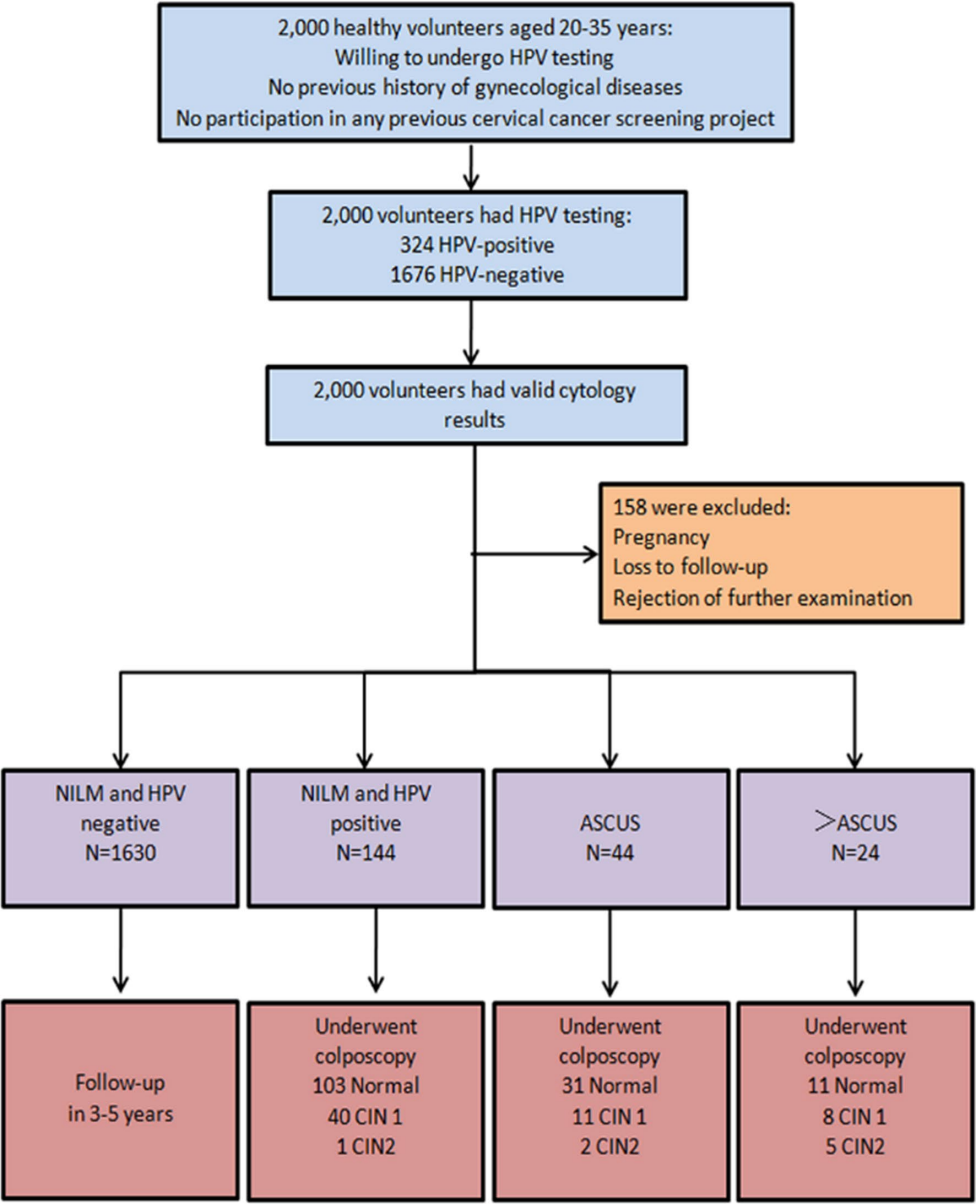
Flowchart of the study protocol

The population eligible for this study were healthy volunteers aged 20–35 years from 8 cities in Fujian (Fuzhou, Fuqing, Nanping, Xianyou, Longyan, Shaowu, Shishi and Jinjiang). The volunteers were recruited through targeted advertisements posted on communities and streets in various regions or on the local social media website. All participants provided informed consent. There was not money or other types of compensation. All women received HPV testing and cytology screening by gynecological practitioners between November 2016 and June 2017. The participants were required to fulfill the following criteria: (1) living in the selected regions, (2) volunteered to join the study and comply with follow-up visits, (3) aged between 20 and 35 years with an active sexual life, (4) no history of cervical treatment or surgery, (5) no immune system diseases, sexually transmitted diseases. The exclusion criteria included the following: washed vulva within 48 h, had sexual intercourse or used drug in vagina within the last 3 days, used antibiotics within one month. There were 2000 women aged 20–35 years who volunteered to participate in the study. The average period was 40.6 ± 10.21 days from study enrollment until the biopsy. A flowchart of the study protocol is shown in Fig. 1. The research protocol and all versions of the study documents were approved by the Hospital Ethics Committee of Fujian Provincial Maternity and Children's Health Hospital, affiliated hospital of Fujian Medical University (NO. 2016-019).

### PCR-RDB HPV test

Cervical cells collection, storage method and the PCR-RDB HPV test (Yaneng Biotech) were illustrated in our prior study [7] and are briefly described as follows. HPV genotyping was performed by hybridization and RDB on the strips fixed with 23 different type-specific probes, including 18 HR-HPV types (16, 18, 31, 33, 35, 39, 45, 51, 52, 53, 56, 58, 59, 66, 68, 73, 82, and 83) and 5 Low-risk human papillomavirus (LR-HPV) types (6, 11, 42, 43, and 81). The blue spots on the strip could be judged as positive by the naked eye.

### Liquid-based cytology

All specimens were collected by clinical Physicians who are licensed as professional physicians, Physicians are trained in specimen collection skills. The cytological specimens were blinded and evaluated independently from the results of the other assays by two experienced cytopathologists. If the diagnosis was different, the cervical samples were reviewed again and a consensus diagnosis was obtained.

According to previous study reports [8], at high magnification, the squamous epithelial cells are covered with gram-negative coccidiobacteria, especially along the edges of the cell membrane, which have a clue-like appearance, known as clue cells. Clue cells accounted for more than 20% of the whole epithelium in the film, that is, clue cells were positive. BV was diagnosed if clue cells were observed (20% more than epithelial cells). A total of 20 representative fields containing at least 10 epithelial cells were randomly selected and examined under 40× magnifications. The smear was considered positive for BV when at least two clue cells were found per field. The cytological specimens were blinded and evaluated by two experienced cytopathologists.

### Histology

Women who were HPV-positive and/or had an abnormal cytological result (with a grade higher than atypical squamous cells of undetermined significance (ASC-US) were referred for colposcopy and punch biopsy. Cervical biopsy specimens were then histologically examined and classified according to the CIN system. For women with negative results for cytology and HPV tests at a primary round, as the disease status, it was assumed that no new disease would be observed until further histology was received.

### Statistical analysis

All confidence intervals (CIs) were exact binomial CIs. Chi-square test, Fisher's exact test, and logistic regression were performed. All data analyses were performed using SPSS 22.0 (IBM, Chicago, IL, USA). For all analyses, *P* values were two-sided, and statistical significance was accepted if the *P* value was less than 0.05.

## Results

**Fig. 2:**
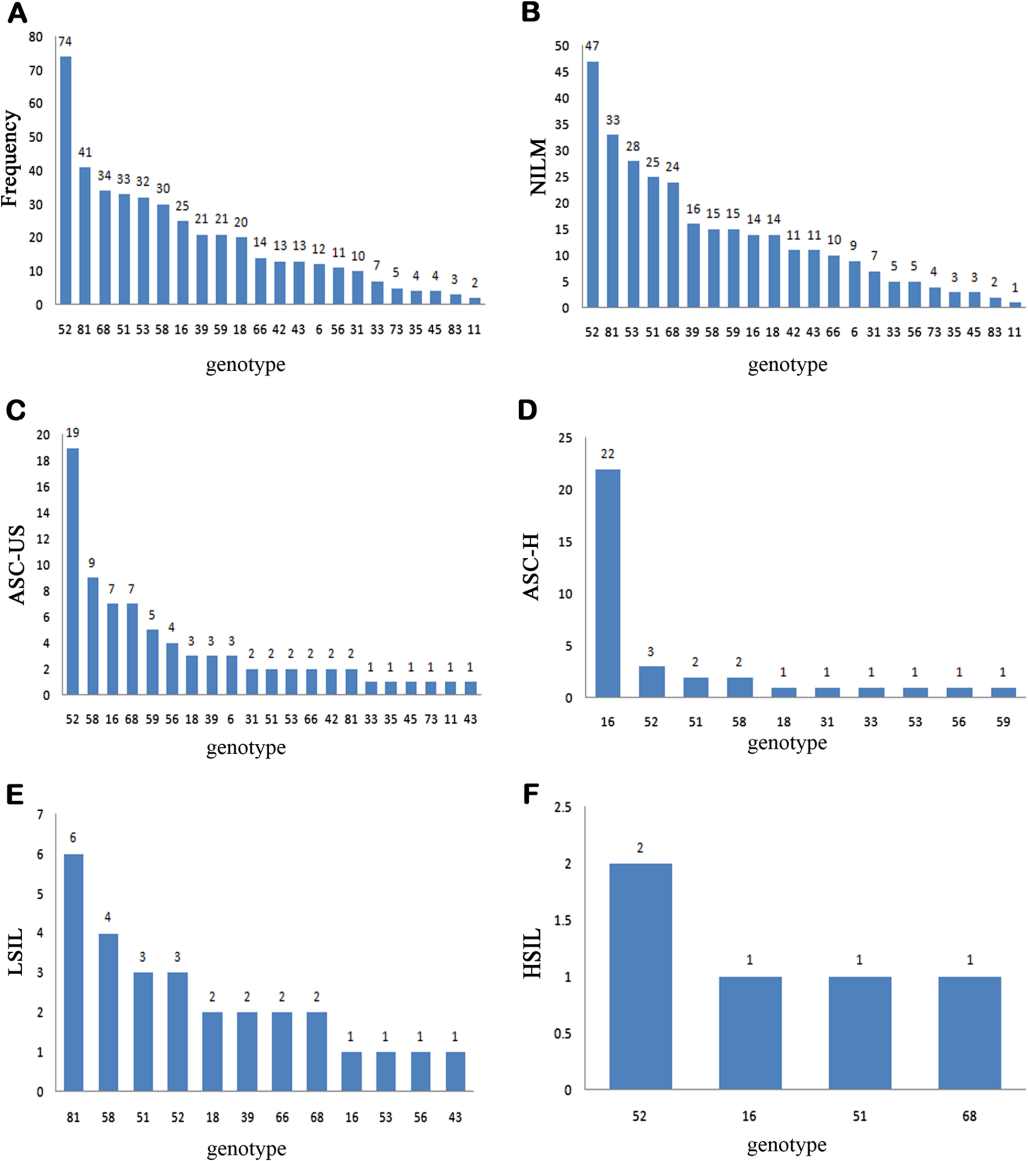
Prevalence of infections with different HPV genotypes in different cervical lesions among Chinese women aged 20–35 years in Fujian. **A** HPV genotype in HPV-positive women. **B**–**F** The blue bars indicate HPV genotypes in HPV-positive women with NILM, ASC-US, ASC-H, LSIL and HSIL. *HPV* human papillomavirus, *NILM* negative for intraepithelial lesion or malignancy, *ASC-US* atypical squamous cells of undetermined significance, *ASC-H* atypical squamous cells, cannot exclude high-grade squamous intraepithelial lesion, *LSIL* low-grade squamous intraepithelial lesion, *HSIL* high-grade squamous intraepithelial lesion

This study cohort consisted of 2000 women who were subject to HPV testing and cytology screening. The average age of the participants in this study was 29.70 ± 3.266 years (range: 20–35 years). According to the results, the overall HPV infection rate in this population was 16.2% (324/2000). In addition, 13.7% (273/2000) of the population was positive for HR-HPV, and 3.8% (76/2000) was positive for LR-HPV. The rate of mixed infection (both HR-HPV and LR-HPV infections) was 1.3% (25/2000). In the HPV infected population, HPV-52 was the most prevalent genotype. The HPV prevalence in different cervical lesions was performed in Fig. 2. Overall, HPV types targeted by bivalent HPV vaccine (HPV-16/-18) were 13.9% (45/324, 95% CI 10.12–17.65), by quadrivalent vaccine (HPV-6/11/16/18) were 17.9% (58/324, 95% CI 13.73–22.08), by nonavalent vaccine (HPV-6/11/16/18/31/33/45/52/58) were 50.0% (162/324, 95% CI 44.56–55.44) in HPV positive women.

**Fig. 3:**
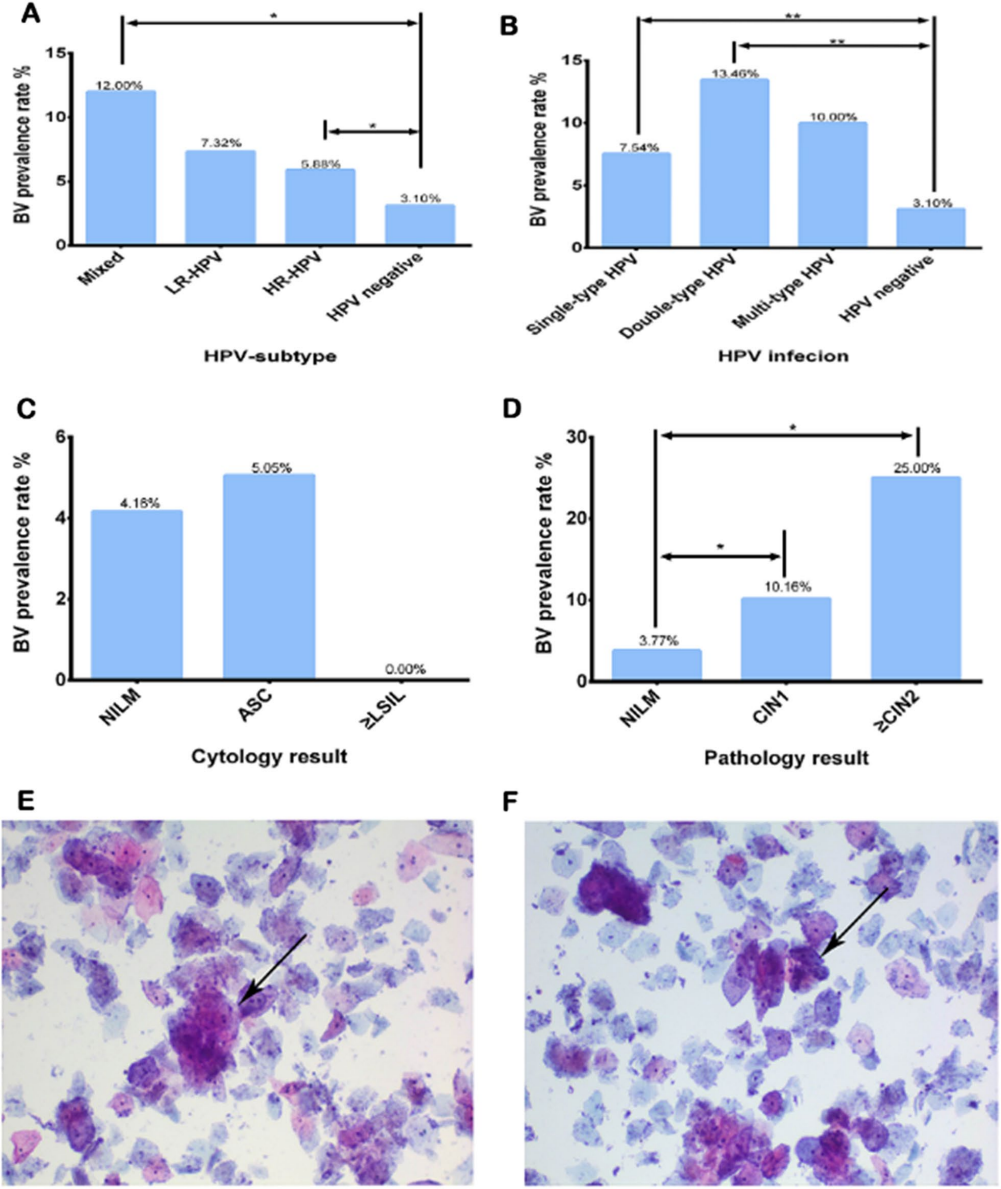
The rate of BV infection (n = 1842). **A** BV infection rate in different HPV-subtypes. **B** BV infection rate in groups with different HPV infections. **C** BV infection rate in groups with different cytology results. **D** BV infection rate in groups with different pathology results. **E** Showed the clue cells of a partially BV-infected individual in HPV-positive groups. **F** Showed the clue cells of a partially BV-infected individual in HPV-negative groups. The arrow pointed at the clue cells. Mixed including LR-HPV and HR-HPV. ASC including ASCUS and ASC-H. ≥ LSIL including LSIL, HSIL and AGC. *P < 0.05, **P < 0.001

In this study, the BV prevalence in different HPV-subtype infections was performed in Fig. 3. Compared with HPV-negative individuals, BV prevalence was higher in the HR-HPV (*P* < 0.001) and mixed-infection groups (*P* = 0.044). However, there was no difference between the LR-HPV and HR-HPV groups regarding BV prevalence (*P* = 0.76). There was no statistically significant difference in BV prevalence between women with single-type HPV infection and multi-type HPV infection (*P* = 0.23). BV was and HPV were more commonly associated with each other. HPV prevalence risk assessment was as follows: HPV-51 (odds ratio [OR]: 5.35; 95% CI 2.05–13.96) and HPV-52 (OR: 2.59; 95% CI 1.11–6.08) (Table 1).

**Table 1:** Association between various subtypes of HR-HPV and bacterial vaginosis (n = 2000)

HR-HPV genotype		No. of women	Odd ratio	95% CI	*P*
HPV-16	BV ( −)	13	1.00(R)	–	
	BV ( +)	1	0.62	0.07–5.60	0.67
HPV-18	BV ( −)	11	1.00(R)	–	
	BV ( +)	3	2.41	0.55–10.64	0.25
HPV-31	BV ( −)	5	1.00(R)	–	
	BV ( +)	2	4.65	0.87–24.96	0.07
HPV-33	BV ( −)	4	1.00(R)	–	
	BV ( +)	1	5.25	0.54–51.22	0.15
HPV-51	BV ( −)	19	1.00(R)	–	
	BV ( +)	6	5.35	2.05–13.96	0.001
HPV-52	BV ( −)	21	1.00(R)	–	
	BV ( +)	7	2.59	1.11–6.08	0.03
HPV-53	BV ( −)	24	1.00(R)	–	
	BV ( +)	4	2.89	0.91–9.16	0.07
HPV-56	BV ( −)	4	1.00(R)	–	
	BV ( +)	1	1.58	0.14–18.10	0.72
HPV-58	BV ( −)	12	1.00(R)	–	
	BV ( +)	3	1.84	0.49–6.94	0.37
HPV-59	BV ( −)	14	1(R)	–	
	BV ( +)	1	2.34	0.48–11.35	0.29
HPV-66	BV ( −)	9	1.00(R)	–	
	BV ( +)	1	1.55	0.14–17.59	0.72
HPV-68	BV ( −)	23	1.00(R)	–	
	BV ( +)	1	0.38	0.04–3.69	0.38

R was reference. *HPV* human papillomavirus, *HR-HPV* high-risk human papillomavirus, *95% CI* 95% confidence interval

The study group displayed the following distribution: 93.9% (1878/2000) with normal cytology, 5.0% (99/2000) with ASC-US and ASC-H (atypical squamous cells of undetermined significance and atypical squamous cells, cannot exclude high-grade squamous intraepithelial lesion), 0.9% (17/2000) with LSIL (low-grade squamous intraepithelial lesion), 0.1% (2/2000) with HSIL (high-grade squamous intraepithelial lesion), and 0.2% (4/2000) with AGC (atypical glandular cells). The HR-HPV positivity rate was 10.7% (201/1878) in women with NILM and 58.2% (71/122) in women with abnormal cytological results (*χ*^2^ = 219.91; *P* < 0.001). HR-HPV prevalence was 55.6% (55/99) in participants diagnosed with ASC-US and ASC-H, 82.4% (13/17) in participants with LSIL, 100.0% (2/2) in participants with HSIL, and 25.0% (1/4) in participants with AGC. The prevalence rate in individuals with ASC (including ASC-US and ASC-H) and LSIL was higher than that in the NILM population (all *P* < 0.001). The LR-HPV positivity rate was 3.3% (62/1878) in women with NILM and 11.5% (14/122) in women with abnormal cytological results (*P* < 0.001). In participants with NILM, the BV prevalence was 4.2% (75/1803). Of women with ASC-US and ASC-H, 5.1% (5/99) were diagnosed as BV. No cases of BV were found in women with LSIL, HSIL or AGC. The BV prevalence rate was not statistically associated with specific cytology results (*P* = 0.61).

Overall, 158 participants were excluded because of pregnancy, loss to follow-up or rejection of further examination. The pathology result was used as the diagnostic gold standard. Participants with HPV positive and cytological results with ASC-US or worse were referred for colposcopy and biopsy. The NILM was detected in 190 cases, accounting for 45.9% (190/256) of the participants, besides 64.2% (122/190) was HPV positive. CIN 1 was found in 59 cases, accounting for 23.1% (59/256) of the participants. Additionally, CIN 2 was detected in 8 cases, accounting for 3.1% (8/256) of the participants. No cases of CIN 3 or cancer were detected. The proportion of HR-HPV infection was 76.4% (110/1775) in participants with cervicitis or NILM, 93.2% (55/59) in participants with CIN 1, and 100.0% (8/8) in participants with CIN 2 (*P* < 0.001). Cervical lesions were associated with HR-HPV infection, no matter in normal and abnormal cytology results (Table 2). In participants with cervicitis, the BV Positive rate was 3.8% (67/1775). In participants with CIN 1, BV prevalence was 10.2% (6/59). BV account for 25.0% (2/8) in CIN 2. BV prevalence was statistically associated with specific pathological results.

**Table 2:** Compare HPV infection and TCT with pathological diagnosis

Cytology		Histological diagnosis			*P*
		NILM	CIN1	CIN2	
Total (n = 1842)	HPV( +) (n = 193)	129(7.00%)	56(3.04%)	8(0.43%)	< 0.001
	HPV( −) (n = 1649)	1646(89.36%)	3(0.16%)	0(0.00%)	
	Clue cells( +) (n = 75)	67(3.64%)	6(0.33%)	2(0.11%)	0.004
	Clue cells( −) (n = 1767)	1708(92.73%)	53(2.88%)	6(0.33%)	
Normal (NILM)(n = 1774)	HPV( −) (n = 1630)	1630(91.88%)	0(0.00%)	0(0.00%)	Ref
	LR-HPV( +) (n = 16)	15(0.85%)	1(0.06%)	0(0.00%)	0.01
	HR-HPV( +) (n = 116)	80(4.51%)	35(1.97%)	1(0.06%)	< 0.001
	Mix-HPV( +) (n = 12)	8(0.45%)	4(0.23%)	0(0.00%)	< 0.001
Abonormal (≥ ASC-US) (n = 68)	HPV( −) (n = 19)	16(23.53%)	3(4.41%)	0(0.00%)	Ref
	LR-HPV( +) (n = 3)	3(4.41%)	0(0.00%)	0(0.00%)	> 0.999
	HR-HPV( +) (n = 41)	20(29.41%)	15(22.06%)	6(8.82%)	0.03
	Mix-HPV( +) (n = 5)	3(4.41%)	1(1.47%)	1(1.47%)	0.23

Clue cells > 20% was positive; ≥ ASCUS, including ASC-US, ASC-H, LSIL, HSIL and AGC. *P* value was obtained from Fisher's exact test. LR-HPV positive was LR-HPV positive only. HR-HPV positive was HR-HPV positive only. Mix-HPV positive was both LR-HPV positive and HR-HPV positive. HPV positive were mix-HPV positive, LR-HPV positive and HR-HPV positive. *CIN* cervical intraepithelial neoplasia, *HPV* human papillomavirus, *NILM* negative for intraepithelial lesion or malignancy, *TCT* thinprep cytologic test

## Discussion

There are limited epidemiological data simultaneously evaluating the relationship between HPV prevalence and BV prevalence and their effect on cervical lesions among women aged 20–35 years in the Fujian province. In this study, the population aged 20–35 years, showed a relatively high HPV prevalence, but the rate of abnormal cervical lesions was low, which highlights the importance of HPV vaccines. In addition, a previous study suggested that the mortality rate of cervical cancer among young urban women increased by 4.1 percent per year in China, according to rough statistics [9]. However, current cervical cancer screening guidelines focus primarily on women older than 30 years. Therefore, the rate of missed cervical cancer diagnosis in young women under 30 years should receive more attention. According to our previous study [10], most women aged 20–35 years are willing to receive and pay for the HPV vaccine. Therefore, it is necessary to investigate the distribution of HPV and BV in women of this age group for the future application of cervical cancer vaccines in Fujian.

Several epidemiological studies have suggested a substantial difference in HPV prevalence and genotype distribution among different regions [11, 12], varying from 9.2% to 18.3%. In our study, the HPV prevalence rate was 16.2%, which is similar to the results of previous studies. However, the prevalence of HPV was 7.2% in Southeast Asia [13]. Compared with neighboring countries, the HPV prevalence rate in Fujian was higher, possibly because of the lack of HPV vaccine implementation and economic underdevelopment.

In our study, HPV-52 was the most common HR-HPV, consistent with the previous studies [14]. The next most frequent types were HPV-81, -68, -51 and -53. In other regions of China, the result was different. Ding et al. [14] suggested that the most common HPV genotypes were HPV-52, -16, -81, -58, and -18. A study in four large cities and four rural areas in China demonstrated HPV-16 to be the most common type, followed by HPV-58 and HPV-18 [15]. All of these data were based on a lifetime age range, and the age-specific data were limited. A study conducted by Li et al. [16] was performed among young women, but the study population was all from Western China. Recently, a study in Fujian province suggested that HPV-16, -52, -58, -43, and -18 were the most common types [7]. However, this study was performed in a hospital population. The HPV distribution in a community population remains unknown. Our study is unique as a relatively large study investigating the HPV genotype distribution among women aged 20–35 years in the community, showing that the most common HR-HPV infection types were HPV-52, -68 and -51, which carry a relatively high risk for cervical lesions and even cervical cancer.

In Fujian, as for HPV vaccine was approved for a short time, many challenges remain in the development and application of the HPV vaccine. In our study, the HPV vaccine targeted genotypes were only 13.9% (45/324) in the bivalent HPV vaccine (HPV-16/-18) and 17.9% (58/324) in the quadrivalent vaccine (HPV-6/-11/-16/-18). Although the nonavalent vaccine (HPV-6/-11/-16/-18/-31/-33/-45/-52/-58) targeted 50.00% (162/324) of the HPV genotypes, this vaccine has not been approved in Fujian. Thus, in this population, the introduction of Gardasil-9 should be encouraged. In addition, because the current HPV vaccines are both prophylactic and not effective in women with HPV infection, cervical cancer screening programs are necessary for early detection of precancerous cervical lesions and to treat HPV infection to reduce the rate of cervical cancer. Our study may provide a basis for formulating cervical cancer prevention strategies in the Fujian region.

Currently, the association between BV and cervical HPV infection identified in several studies remains controversial [17]. When analyzing the association between BV and cervical HPV infection, our study showed that HR-HPV infection was significantly correlated with BV. Our results were consistent with those of a previous study [18]. Eun et al. [19] suggested that persistent HPV infection may change the vaginal milieu in women. A study conducted by Guo et al. [18] indicated that BV may delay HPV infection clearance. Another study suggested that BV can disrupt local immune defense [20]. The reasons for the association between BV and cervical HPV remain controversial and unknown. Furthermore, it is unknown how HPV infection or BV disrupts the vaginal microenvironment balance. Vaginal microenvironment disorder has been associated with increased levels of proinflammatory cytokines and chronic inflammation at mucosal sites. Women with BV expressed increased levels of cytokine interleukin (IL)-1β and decreased levels of IL-17 [21, 22]. It has also been reported that G. vaginalis can secrete sialidase while elevated sialidase concentration was associated with increased risk for cervical lesion [23]. This type of immune activation needs to be confirmed in further studies with rigorous experimental precision.

Up until now, there are few studies focus on the correlation between BV and specific HPV genotypes. According to the results of this study, BV was found to be related to HPV-51, -52 infections. Previews study in Africa suggested that BV associate with HPV-58 [24]. Obviously, the epidemiology of HPV and genital tract infection is different in each region, and the founding of this study may guide the HPV vaccine protecting patients with recurrent BV. Our study emphasizes the importance of treating BV for preventing HPV infection, particularly for HR-HPV infections.

However, this study also has some limitations. First, BV can be diagnosed by the Amsel's criteria, Nugent scoring system, Hay-Ison score [25], and Emerging molecular and functional diagnostic methods [26]. Although Amsel’s criteria is considered as the “gold standard” of BV clinically diagnosis, the method is often subject to interobserver variability as the assessment of the diagnostic criteria depends on the observer's skill and experience. So the specimens were blinded and evaluated by two experienced laboratory physicians. Secondly, our study only focused on BV, but other sexually transmitted infections such as *Neisseria gonorrhoeae*, *Trichomonas vaginosis* or *Mycoplasma genitalium* were not included. Nowadays, there is growing research into the relationship between other sexually transmitted infections (STI) and HPV. 11.4% of the women had at least one STI pathogen, the most common being Chlamydia trachomatis (6.7%) [27]. Chlamydia trachomatis and HPV-16, HPV-51, HPV-52 was most frequently identified in co-infections [28]. The mechanism of interaction between HPV and other pathogens is unclear. Further investigations should be conducted to explore the interaction between HPV and other pathogens, and the sample size should be expanded.

## Conclusion

This study observed a relatively high HPV infection rate in 20–35 years women in Fujian. The high prevalence of HPV genotypes targeted by nonavalent vaccines suggested that the introduction of nonavalent vaccine may benefit a large number of women at high risk for HPV infection. Meanwhile, BV was found to be related to HPV-51, -52 infections and cervical lesions, suggesting that attention should be paid to the prevention, discovery and proper management of BV.

## Data Availability

The datasets used and/or analyzed during the current study are available from the corresponding author on reasonable request.

## Abbreviations

BV: Bacterial vaginosis
TCT: ThinPrep cytologic test
HPV: Human papillomavirus
HR-HPV: High-risk human papillomavirus
LR-HPV: Low-risk human papillomavirus
PCR-RDB HPV test: PCR-reverse dot blot human papillomavirus genotyping
ASC-US: Atypical squamous cells of undetermined significance
ASC-H: Atypical squamous cells of undetermined significance and atypical squamous cells, cannot exclude high-grade squamous intraepithelial lesion
AGC: Atypical glandular cells
CIN: Cervical intraepithelial neoplasia
LSIL: Low-grade squamous intraepithelial lesion
HSIL: High-grade squamous intraepithelial lesion
NILM: Negative for intraepithelial lesion or malignancy
STI: Sexually transmitted infections
